# Unidirectional transitions in nectar gain and loss suggest food deception is a stable evolutionary strategy in *Epidendrum* (Orchidaceae): insights from anatomical and molecular evidence

**DOI:** 10.1186/s12870-018-1398-y

**Published:** 2018-09-04

**Authors:** Poliana Cardoso-Gustavson, Mariana Naomi Saka, Edlley Max Pessoa, Clarisse Palma-Silva, Fabio Pinheiro

**Affiliations:** 1 Instituto de Botânica, Núcleo de Pesquisa do Orquidário do Estado, São Paulo, SP 04301-902 Brazil; 2 Departamento de Botânica, Instituto de Biociências, Universidade Estadual Paulista, Rio Claro, SP 13506-900 Brazil; 3 Centro de Ciências Biológicas, Departamento de Botânica, Universidade Federal de Pernambuco, Recife, PE 50670-420 Brazil; 4 Departamento de Biologia Vegetal, Instituto de Biologia, Universidade Estadual de Campinas, Campinas, SP 13083-862 Brazil

**Keywords:** Character reconstruction, Cuniculus, Evolution, Nectar, Nectary anatomy, Orchidaceae, Osmophores, Phylogeny, Rewardless species

## Abstract

**Background:**

Nectar gain and loss are important flower transitions observed in angiosperms, and are particularly common in orchids. To understand such transitions, the availability of detailed anatomical data and species-level phylogenies are crucial. We investigated the evolution of food deception in *Epidendrum*, one of the largest orchid genera, using genus phylogeny to map transitions between nectar gain and loss among different clades. Associations between anatomical and histochemical changes and nectar gain and loss were examined using fresh material available from 27 species. The evolution of nectar presence/absence in *Epidendrum* species was investigated in a phylogenetic framework of 47 species, using one nuclear and five plastid DNA regions available from GenBank and sequenced in this study.

**Results:**

The presence or absence of nectar was strongly associated with changes in the inner epidermal tissues of nectaries. Nectar-secreting species have unornamented epidermal tissue, in contrast to the unicellular trichomes found on the epidermis of food deceptive species. Bayesian tests confirmed that transitions occurred preferentially from nectar presence to nectar absence across the *Epidendrum* phylogeny. In addition, independent nectar loss events were found across the phylogeny, suggesting a lack of constraint for these transitions.

**Conclusions:**

Ornamented nectaries may play an important role in the deceptive pollination strategy by secreting volatile organic compounds and providing tactile stimuli to pollinators. The recurrent and apparently irreversible pattern of nectar loss in *Epidendrum* suggests that food deception may constitute an alternative evolutionarily stable strategy, as observed in other orchid groups.

**Electronic supplementary material:**

The online version of this article (10.1186/s12870-018-1398-y) contains supplementary material, which is available to authorized users.

## Background

Floral rewards are one of the most conspicuous adaptations of angiosperms, playing a central role in the attraction and maintenance of pollinators [1]. However, a large number of species deceive their pollinators by mimicking signals that are associated with food, sexual partners, or oviposition sites. The loss of flower rewards has several biological implications for plant populations. In general, pollinators avoid rewardless flowers by visiting distant plants, enhancing interpopulation genetic exchange, and consequently reducing the genetic structure of populations [2] and the levels of geitonogamy [3, 4]. Studies that added artificial nectar to rewardless flowers observed a significant increase in fruit set and self-pollination [5]. According to Johnson et al. [6], selection toward rewardless species is favored when pollinators are common, and nectar production is selected when pollinators are scarce.

Orchids deceive their pollinators in several ways, but generalized food deception is the most common strategy [5]. Despite the lack of nectar in many species, nectaries are widespread in the family, exhibiting extensive variation in morphology, anatomy and position. Nectaries can be associated with the lip callus, lip spur, column, or with a nectar spur formed by the fusion of lip, column, and sepal margins [7, 8]. In several genera of subtribe Laeliinae, there is a nectary-like structure below the column called a cuniculus. The cuniculus is a floral tube that originates at the fusion of the lip claw and column and penetrates the pedicel between the ovary and the perianth tube (Additional file 1: Figure S1). The presence of a cuniculus is often associated with *Lepidoptera* pollination, which is commonly found in *Epidendrum* L. (reviewed by [9]). Few anatomical studies have investigated this structure in depth (but see [10]), and whether nectar is present within the structure is unknown in many species. In *Epidendrum*, nectar has been detected only in species showing swollen cuniculi, in which the presence of copious nectar is evident [9]. Thus, rewarding and rewardless *Epidendrum* species are distinguishable only through observing free nectar availability in floral nectaries [11, 12].

To understand the evolution of food deception, detailed anatomical data have been mapped into phylogenies, revealing the transitions between nectar gain and loss across different clades. A lack of floral nectar is a potential ancestral trait in the Orchidaceae due to the lack of nectar in the ancestral genera *Apostasia* and *Neuwiedia*, which offer pollen as a reward [13]. Within the family, independent and reversible transitions have been observed in different clades. There are groups for which food deception is the ancestral state, as in the tribe Orchideae [14], and cases in which nectar reward evolved secondarily from food deception, as observed in the genera *Disa* [15] and *Anacamptis* [16]. A gain or loss of nectar may result in different anatomical responses. Transitions may be associated with deep structural changes in nectaries, as observed in the subtribe Orchidinae [17], or may result in the pattern observed in *Disa*, in which little epidermal change has been observed between nectar-producing and nectarless species, suggesting that subcellular modifications may control nectar production [18].

In the present study, we investigated the evolution of nectar reward in the genus *Epidendrum*, which, with approximately 1500 species, is considered one of the largest plant genera of the neotropics [19]. The presence/absence of nectar in *Epidendrum* species was inferred based only on visual inspection of nectaries, and detailed histochemical and/or anatomical analyses have been rarely reported in this genus [12, 20]. Due to the apparent lack of nectar, many authors have considered food deception to be widespread within *Epidendrum* (reviewed by [9]). Indirect evidence gathered from different data sources has been interpreted as collective evidence indicating the presence of a food deceptive strategy. The short visit times of pollinators that abandon the inflorescence after visiting a single flower [11], low fruit set [12], and the absence of nuclear genetic structure among populations [9] may indicate the existence of food deception in the analyzed species. According to [2], food deception in orchids tends to reduce the time spent by pollinators in the same inflorescence, decreasing the levels of geitonogamy and fruit set. Despite the putative lack of nectar, no *Epidendrum* species have been submitted to detailed anatomical analyses aimed at exploring the structure and nature of the epidermal tissue inside the nectary, or cuniculus.

Due to the large size of the genus, we focused our study on a smaller clade, the group *Amphyglottium* (sensu [19]), which has a previous phylogenetic hypothesis based on amplified fragment length polymorphism (AFLP) fingerprinting and plastid markers [21]. New nuclear and plastid regions were sequenced to obtain a robust phylogenetic hypothesis for the group and to study the divergence time of main clades within the subgenus. Detailed anatomical observations on nectaries from all species were also included, using stereomicroscopy and scanning electron microscopy (SEM). The primary aim of the present study was to use a dated molecular phylogeny to study the evolution of nectar reward in the group *Amphyglottium* and selected *Epidendrum* species to identify nectar-producing and nectarless species. The following specific questions were addressed: (1) What is the timing and tempo of species diversification in the group *Amphyglottium*? (2) How many times have nectar and specific nectary types evolved in the group? (3) Are anatomical changes associated with the gain and loss of nectar production? Overall, this study supported the recurrent and unidirectional evolution of nectar loss in *Epidendrum* nectaries, which differ in anatomical features compared with nectaries from nectar-secreting species.

## Results

### Structure and identification of the cuniculus glands

**Fig. 1:**
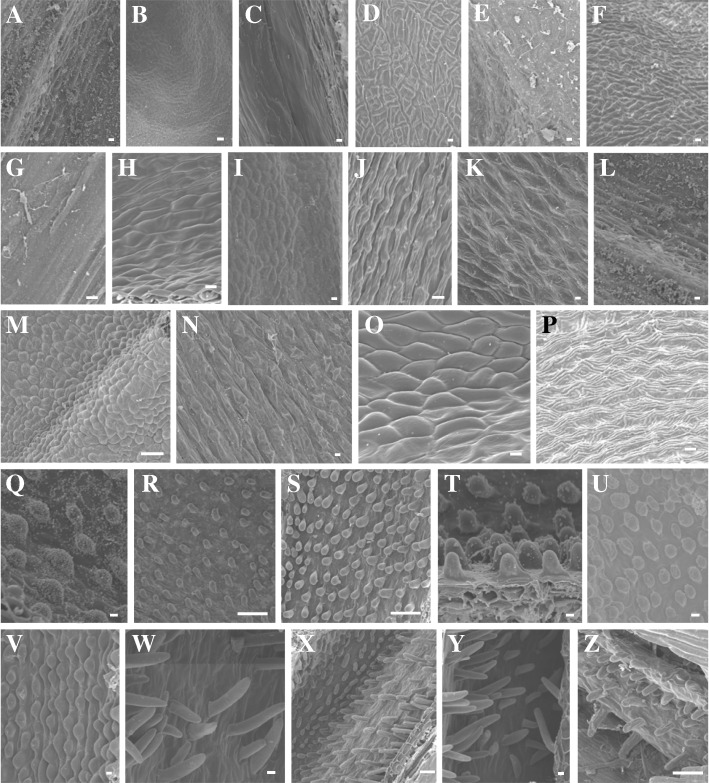
Scanning electron micrographs showing the glandular epidermis of the cuniculus in selected *Epidendrum* species. **a**–**g** Ordinary nectary epidermis. **a** *E. armeniacum.* **b** *E. cristatum.* **c** *E. densiflorum*. **d** *E. orchidiflorum.* **e** *E. nocturnum.* **f** *E. vesicatum*. **g** *E. viviparum.* **h**–**p** Short trichomes. **h** *E. calanthum.* **i** *E. campestre.* **j** *E. ciliare.* **k** *E. coronatum.* **l** *E. ibaguense.* **m** *E. incisum*. **n** *E. purpureum.* **o** *E. radicans.* **p** *E. robustum.* **q**–**v** Medium trichomes. **q** *E. cochlidium*. **r** *E. denticulatum.* **s** *E. flammeus*. **t** *E. puniceoluteum.* **u** *E. secundum.* **v** *E. xantinum.* **w**–**z** Long trichomes. **w** *E. anceps.* **x** *E. cinabarinum.* **y** *E. fulgens.* **z** *E. rigidum.* Scale bars = 10 μm (**a**–**l**, **n**–**q**, **t**-**w**, **y**) and 100 μm (**m**, **r**, **s**, **x**, **z**)

**Fig. 2:**
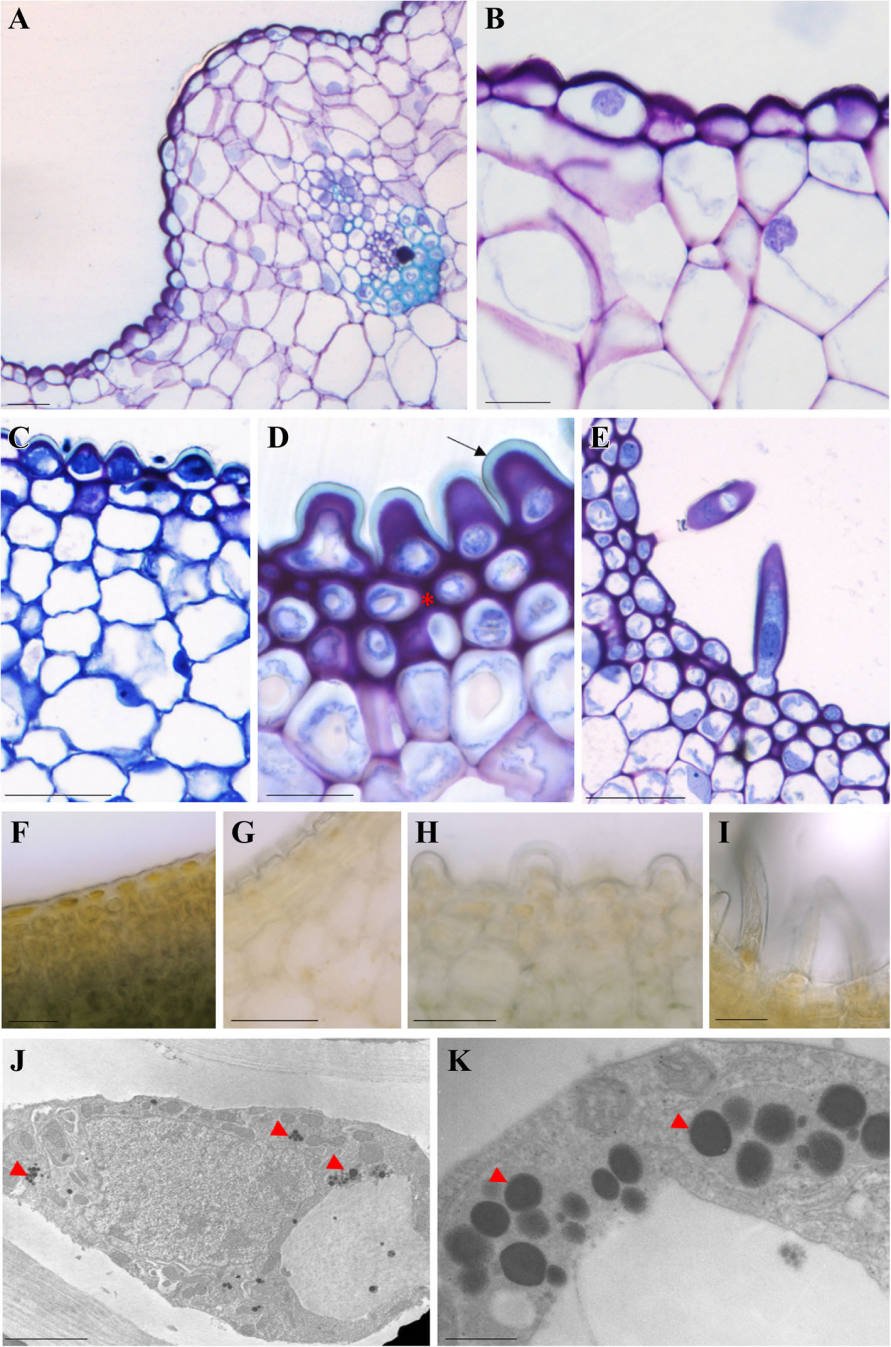
Cellular aspects of cuniculus glands in selected *Epidendrum* species. **a**–**e** Structural aspects stained with toluidine blue O. **f**–**i** Sections stained with Fehling’s reagent for reducing sugars. **j**–**k** Ultrastructure of the glandular trichomes. **a**, **b**, **f** Ordinary nectary epidermis in *E. vesicatum* (**a** and **b**); positive staining indicative of reducing sugars in the nectary epidermis and parenchyma (**f**). **c**–**e**, **g**–**k** Unicellular glandular trichomes (osmophores). Small trichomes in *E. ibaguense* (**c**) and *E. ciliare* (**g**)*.* Medium trichomes in *E. flammeus* (**d**) and *E. cochlidium* (**h**, **k**)*.* **e**, **i**–**j** Long trichomes in *E. fulgens*. Note the thick cuticle (arrow in **d**), the adjacent collenchyma tissue (* in **d**), and the absence of reducing sugars inside the glandular trichomes, regardless of their size (**g**–**i**). Lipophilic droplets (red arrowheads in **j** and **k**) are observed in the protoplasts of glandular trichomes. Scale bars = 50 μm (**a**–**i**) and 0.5 μm (**j**, **k**)

*Epidendrum* species have a cuniculus (a glandular chamber) positioned along the stylar canal between the perianth tube and the pedicellate ovary (Additional file 1: Figure S1). The inner epidermis of the cuniculus is always glandular, and its lumen varies among species and is wider in nectar-producing structures. Here we described the glands at secretory phase in flowers at anthesis. Four glandular morphologies (Figs. 1 and 2) were identified: (i) an almost uniform epidermis lacking trichomes, stomata, or cuticle disruption (designated “ordinary epidermis” Figs. 1a–g, 2a, b) and three sizes of glandular unicellular trichomes; (ii) short trichomes, composed of secretory papillae (Figs. 1h–p, 2c); (iii) medium trichomes (Figs. 1q–v, 2d); and (iv) long trichomes (Figs. 1w–z, 2e), which were at least twice as long as the medium ones.

Almost all nectar-producing flowers produced an exudate profuse enough to be tested with glucose strips. The exudates of *E. densiflorum*, *E. mirmecophorum*, *E. nocturnum*, and *E. vesicatum* tested positive for reducing sugars. The cuniculi of these species, as well as the remaining ones of the ordinary epidermis type, showed positive results for reducing sugars in the vacuoles of the epidermis and parenchyma (usually three cell layers), and were therefore identified as floral nectaries (Fig. 2f).

The glandular trichomes had thick cuticles and cell walls, regardless of their size. In addition, a conspicuous collenchyma tissue composing the one-to-three-layered subepidermal region was noteworthy (Fig. 2c–e). The absence of reducing sugars in all glandular trichomes and collenchyma tissue was also remarkable (Fig. 2g–i). Conversely, the occurrence of lipophilic droplets in the protoplast of the trichomes, observed under TEM (Fig. 2j, k), indicated that these glands may act as osmophores. In summary, all unornamented cuniculi showed signs of nectar secretion, whereas no signs of nectar were observed in ornamented cuniculi. This pattern was used to map the ancestral states in the phylogenetic tree and to infer the presence of the food deception strategy in the genus *Epidendrum* (see below).

### Divergence times of *Epidendrum* clades and species

**Fig. 3:**
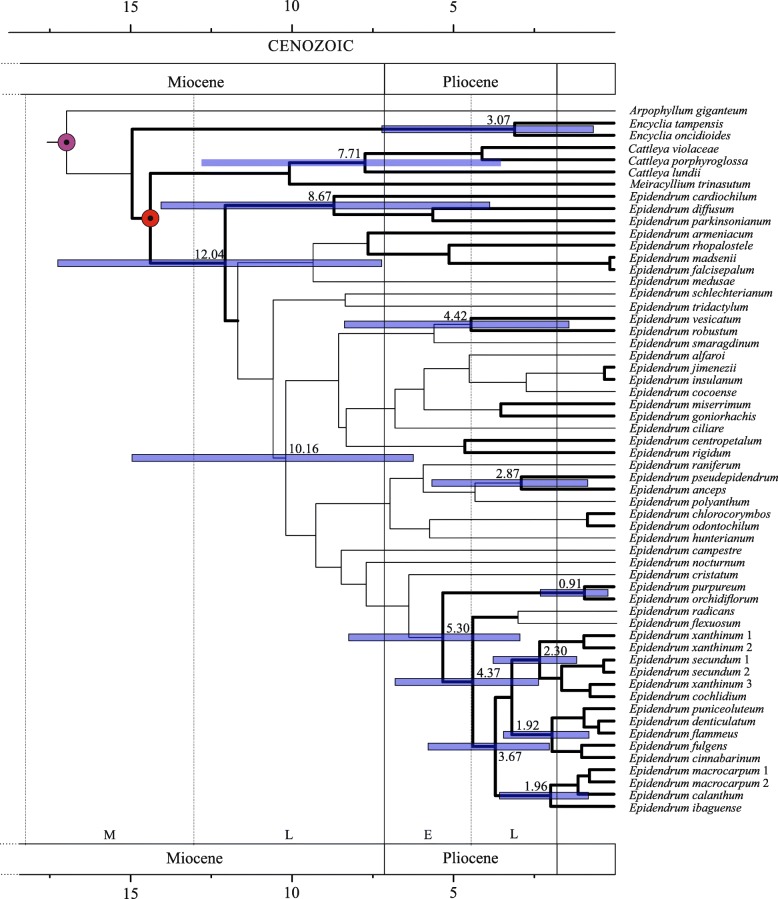
Time-calibrated tree of *Epidendrum* based on one nuclear and five plastid regions estimated with BEAST. *Arpophyllum*, *Cattleya*, *Encyclia*, and *Meiracyllium* species were used as outgroups. Thin branches indicate posterior probabilities lower than 0.9. Numbers at nodes indicate median ages in millions of years (Ma). Node bars indicate the 95% HPD lower and upper bounds in Ma. Circles indicate age-constrained nodes; the purple circle indicates the calibration point for Laeliinae, and the red circle indicates the calibration point at the *Cattleya* + *Epidendrum* + *Meiracyllium* clade*.* GenBank accession numbers of nuclear (ITS) and plastid (matK, rbcL, trnL–trnF, trnT–trnL, and rpl32–trnL) regions are provided in the Additional file 4: Table S1

The Bayesian inferences for the first (primary calibration) and second (secondary calibration) analyses based on the combined data sets resulted in largely congruent topologies that proved stable in repeated runs. Tracers found stabilized likelihoods, smooth distributions, and high effective sample sizes for all parameters of all Bayesian runs. Bayesian inferences of the partitioned data sets (primary calibration = matK and rbcL; secondary calibration = matK, rbcL, trnL–trnF, trnT–trnL, rpl32–trnL, and ITS) produced the most resolved and well-supported trees (Additional file 2: Figure S2, Fig. 3).

The maximum credibility tree for the fossil-calibrated relaxed molecular clock analysis of the family Orchidaceae is shown in Additional file 1: Figure S2. Support values for the different clades and branches were high, with only a few BPP values below 0.90. Our age estimates indicate that the subfamilies Orchidoideae and Epidendroideae started to diversify in the Eocene, 51.6 Ma and 42.2 Ma, respectively (Additional file 1: Figure S2). The diversification of Higher Epidendroids appears to have started during the Oligocene, approx. 34.5 Ma. Our age estimates also indicate that the subtribe Laeliinae diversification started during the Early Miocene, approx. 20.1 Ma.

The maximum credibility tree for the calibrated relaxed molecular clock analysis of the Higher Epidendroids, focusing on the subtribe Laeliinae and the genus *Epidendrum*, is shown in Fig. 3. The support values were high for the clade including *Encyclia*, *Cattleya*, and *Meiracyllium*, with BPP > 0.90. All *Epidendrum* species clustered together, with high support values (PP > 0.90), supporting the monophyly of the genus. However, most clades within the genus showed low resolution, with BPP < 0.90, except group *Amphyglottium*. High support values were also observed within group *Amphyglottium*, and only the group formed by *E. radicans* and *E. flexuosum* showed support values below 0.90 BPP. The informal clades recovered by Pinheiro et al. (2009a) were also present in our analysis. Four main clades were observed, the Andean clade (*E. calanthum*, *E. ibaguense*, and *E. macrocarpum*), which is sister to the *Tuberculata* (*E. cochlidium*, *E. secundum*, and *E. xanthinum*) and Atlantic clades (*E. cinnabarinum*, *E. denticulatum*, *E. flammeus*, *E. fulgens*, and *E. puniceoluteum*), and the clade formed by *E. orchidiflorum* and *E. purpureum*, which is sister to the remaining species (Fig. 3). According to our dating, the genus *Epidendrum* started its diversification during the Late Miocene, approximately 12 Ma. Group *Amphyglottium* started to diversify in the early Pliocene, approximately 5.3 Ma.

### Nectar presence/absence transitions

**Fig. 4:**
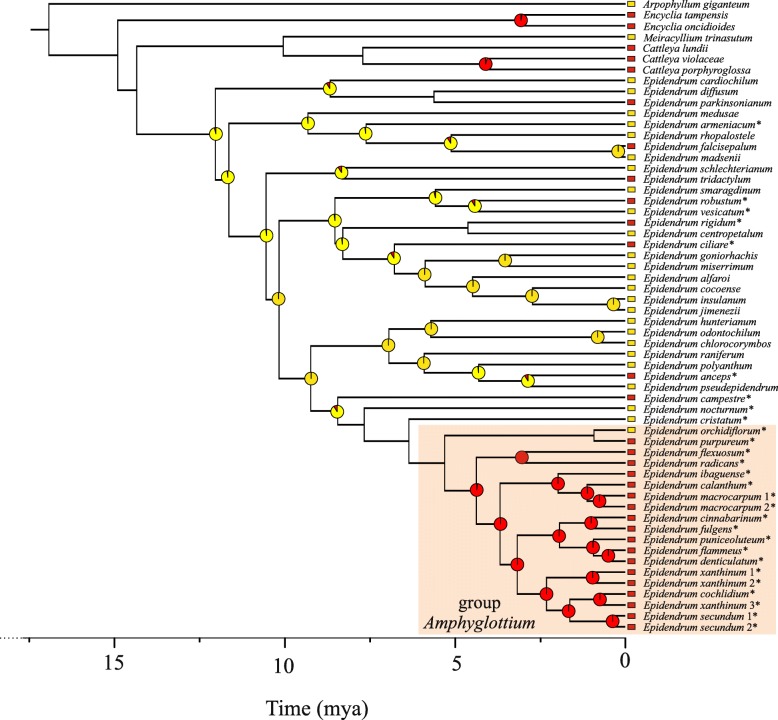
Evolution of nectar presence in 47 *Epidendrum* species based on maximum likelihood. From an ancestor with nectar, rewardless species evolved at least ten times in different clades. Maximum likelihood reconstruction of ancestral states over 100,000 chronograms are summarized on the maximum-clade credibility chronogram. Pie charts at selected nodes summarize the results of the maximum likelihood character optimization analyses. Each chart shows the percentage of trees ≥90% for nectar presence (yellow) or nectar absence (red). Rectangles at branch tips indicate the presence (yellow) or absence (red) of nectar. The group *Amphyglottium* is highlighted in orange. Asterisks indicate species in which nectar presence/absence was inferred in the present study using anatomical analyses

Ancestral state reconstruction suggested unidirectional transitions, with nectar presence as the most common ancestral state of most species (Fig. 4). The number of transitions from NA to NP (minimum 0, maximum 1, average 1.48) was lower than the converse situation (minimum 2, maximum 9, average 5.06). Indeed, MCMC analyses confirmed that the log-likelihood of the restricted model, allowing only transitions from NP to NA (*q*_10_), was significantly better than the unrestricted model or the restricted model allowing only transitions from NA to NP (*q*_01_) (Table 1). Nectar presence was the ancestral character state detected for most *Epidendrum* species, even in clades in which nectarless species were nested with nectar-producing species. The ancestral character state for the group *Amphyglottium* is uncertain, as nectar presence was recovered as the ancestral character state for this clade in only 35% of the trees. However, a different picture was observed within group *Amphyglottium*, in which most species do not produce nectar, and nectar absence was the primary ancestral condition in almost all clades.

**Table 1:** Models testing transitions between nectar presence (NP = 1) and nectar absence (NA = 0) across the *Epidendrum* phylogeny, using the Bayeasian Markov Chain Monte Carlo (MCMC) method, including the harmonic mean of log-likelihoods for MCMC analyses (lnL), the deviance of harmonic means between unrestricted and restricted models (Dev), the probability of NA-to-NP transitions (*q*01), the probability of NP-to-NA transitions *(q*10), the probability of NA at the root of the tree [Root P(0)] and the probability of NP at the root of the tree [Root P(1)]

Model	lnL	Dev^a^	q01	q10	Root P(0)	Root P(1)
MCMC unres.	−42.26		4.92	5.82	0.48	0.52
MCMC 01 = 0	−41.77	**0.98**	0.00	0.06	0.00	1.00
MCMC 10 = 0	−44.93	5.34	0.09	0.00	1.00	0.00

The value in bold indicate the preferred model^a^Decisions over models were taken following Raftery [61]: values > 2 indicate the more complex, unrestricted model should be favored, and values < 2 indicate the more simple, restricted model, should be preferred

We examined a total of 1100 species described by Hágsater et al. in *Icones Orchidacearum* (Additional file 3: Table S2), and a smooth inner epidermal surface was observed in the cuniculus (lack of epidermal unicellular trichomes) of most species. An ornamented cuniculus was found in 113 species (Additional file 3: Table S2), suggesting that approximately 10.3% of *Epidendrum* species do not produce nectar.

## Discussion

The evolution of rewardless species may have several biological implications, such as altering pollinator behavior, patterns of gene exchange, type of reproductive isolation, and rates of speciation. These implications are particularly well known for a few orchid groups, such as subfamily Orchidoideae [2, 3]. The availability of well-sampled phylogenies coupled with detailed anatomical analyses has provided crucial information for understanding how nectarless species evolved [17, 18]. The availability of such informative datasets has been a major challenge in understanding the evolution of rewardless species. In this study, datasets of DNA sequences and nectary anatomy were used to investigate the evolution of rewardless species in *Epidendrum*, mapping the transitions of nectar-producing and nectarless species over time. *Epidendrum* is a monophyletic group that started its diversification during the Late Miocene, likely from a nectar-secreting ancestor (Figs. 3 and 4). Transitions between nectar-secreting and nectarless species occurred independently several times along different time frames. Transitions were significantly unidirectional, with nectar presence the most probable ancestral state for most clades (Table 1). Despite several gains and losses detected along the phylogeny, almost all species from group *Amphyglottium* showed rewardless flowers. Comparing the tissue morphologies of closely related nectar-producing and nectarless species revealed that gain or loss of nectar production was followed by major histological changes (Figs. 1 and 2). Nectar-secreting species exhibited a uniform, unornamented inner epidermis of the cuniculus, with signs of reducing sugars, indicating nectar secretion. In contrast, glandular unicellular trichomes were found in the cuniculi of nectarless species. These trichomes were identified as osmophores and may play an important role in attracting pollinators by signaling a putative reward. The recurrent origin of glandular unicellular trichomes in deceptive species suggests convergent functional evolution, achieved through a widespread absence of morphological constraints associated with nectar losses, as observed in other orchid groups [17, 18].

### The timing of diversification and nectary transitions in *Epidendrum*

The genus *Epidendrum* started its diversification during the Late Miocene (approx. 12 Ma, Fig. 3), coinciding with a period of accelerated Andean uplift [22]. In fact, major shifts in diversification rates occurred in Cymbidieae during the Late Miocene as well, and Pleurothallidinae diversified along altitudinal gradients after major Andean uplifts [23]. Givnish et al. [24] also highlighted the importance of epiphytic habit, pollination by Lepidoptera, and occurrence in tropical cordilleras as drivers of orchid diversification. *Epidendrum* species share most of the traits discussed above, and it is probable that multiple ecological drivers have contributed to the extensive diversification observed in this genus. Most *Epidendrum* species occur along different altitudes in the Andean region as epiphytes in humid forests and are pollinated by Lepidoptera [19].

Our anatomical data clearly associate nectar-producing species with the presence of an unornamented cuniculus, which is a widespread feature in *Epidendrum*. Of the 1100 species descriptions analyzed, only 10.3% showed ornamented cuniculi, which may indicate nectar absence. Thus, contrary to previous reports [9, 19], nectar-producing flowers may constitute the rule rather than the exception in this genus. However, this result needs a careful interpretation and additional confirmation by future studies in the genus, since our sample is very limited when considering the size of the genus *Epidendrum*.

How rewardless flowers contribute to speciation has been a matter of debate in the literature. Inda et al. [14] and Givnish et al. [24] discussed a potential association between increased diversification rates and the occurrence of pollination by deceit in orchids. These authors focused their discussion on different genera from subfamily Orchidoideae, for which the evolutionary outcomes of deceit strategies are well known [2]. In *Disa*, transitions between deceptive and rewarding species did not change speciation rates [15]. Species-level phylogenies have revealed that transitions between deceptive and rewarding pollination systems are not strongly constrained [15, 17]. Thus, rewardless species may evolve in response to certain ecological circumstances, such as pollen limitation, and arise from infrequent pollinator visits. On the other hand, the high levels of species diversity observed in *Epidendrum* may be a result of the high levels of genetic differentiation found in nectar-producing species, compared with rewardless species [3]. Pollinators restrict their foraging range when visiting nectar-producing species, increasing the levels of genetic divergence among plant populations.

Species from group *Amphyglottium* are nested in a well-supported clade that started diverging during the Early Pliocene (5.3 Ma, Fig. 3). Nectar was consistently absent in all species except *E. orchidiflorum* (Fig. 4). Transitions were significantly asymmetric along the phylogeny, with character changes occurring preferentially from nectar presence to nectar absence (Table 1). Thus, nectar-secreting species did not evolve from ancestors lacking reward, suggesting that food deception may constitute an alternative, evolutionarily stable strategy, as observed in other orchid groups [5]. These species are well known for their ability to thrive in harsh environments such as exposed rocky outcrops, sand dunes, and poor soils [9]. Nectar production consumes a significant amount of energy, and nectar loss may represent a selective advantage in stressful habitats, such as those where species from group *Amphyglottium* are found. A similar pattern was observed by Antonelli et al. [25] in a group of *Cattleya* species (formerly *Hoffmannseggella*). All of these species are food deceptive [26], and their occurrence is concentrated in quartzite soils and rocks, which are extremely nutrient-poor. There are additional potential benefits of not producing nectar. For example, the increase in pollinator sharing commonly observed in food deceptive orchids may compensate for strong fluctuations in insect communities [3]. Food deceptive species commonly exhibit weak population genetic structures because pollinators abandon the population when nectarless flowers are visited, transporting pollen over long distances and increasing interpopulation gene exchange [2, 3]. Moreover, hybridization is also commonly observed in food deceptive species from group *Amphyglottium* [4, 27]. Interspecific gene exchange may increase the adaptive responses of species to stressful habitat conditions by exchanging adaptive genes via introgression, as observed in other plant groups [28]. Thus, food deception may constitute an alternative evolutionarily stable strategy in particular ecological conditions [5], enhancing intra- and interspecific gene exchange and decreasing the extinction risk of hybridizing species.

### Anatomical changes in nectar-secreting and nectarless species

The structures of the cuniculus nectaries we observed (Figs. 1 and 2) differed from the descriptions available for the cuniculus of *Brassavola flagellaris* (Laeliinae; [10]) in the resemblance of this structure to the osmophores identified in the *Epidendrum* species lined with unicellular trichomes. The presence of reducing sugars in the exudate, however, undoubtedly identified these glands as nectaries [29]. The nectary type and the absence of trichomes, stomata, or cuticle disruption suggest that nectar is released from nectar-producing cells by cuticular pores [30]. Indeed, the cuticle covering the nectary cells may be completely permeable, allowing the reabsorption of nectar, as observed in *Platanthera chlorantha* [7] and *Epidendrum vesicatum* (Cardoso-Gustavson, pers. obs.).

In Orchidaceae, the osmophores are usually formed by a single layer of epidermal cells or may have secretory trichomes [31, 32]. Here, all trichome-bearing cuniculi, regardless of their size, were identified as osmophores. This identification was based on the presence of many lipophilic droplets in the protoplast, visible by TEM. Lipophilic droplets (also referred to as lipid droplets/bodies or lipophilic inclusions when referring to droplets in the cytoplasm or phenolic compounds in the vacuole, respectively; [33]) are a typical feature of osmophores, being identified in different orchid groups [31, 32, 34]. These droplets can eventually be observed in some nectaries (see [7]), although they may be restricted to specific stages of secretion [35]. Nectaries comprising unicellular trichomes resembling the *Epidendrum* osmophores were described in *Disa* (Disinae; [18]). Indeed, glands were involved in the evolution of pollinating systems in orchid groups [36], constituting the fundamental functional element in the transition from nectarless to nectar-producing flowers [37].

The occurrence of lipophilic droplets in the protoplast can be directly associated with the low polarity of the volatile organic compounds (VOC) [38] composing the floral fragrance emitted by flowers. Indeed, among the species we analyzed, the only one with an available floral VOC profile was the nectarless moth-pollinated *Epidendrum ciliare* [39], in which the occurrence of terpenoids was remarkable. The occurrence of terpenoids has been associated with lipophilic droplets in the cytoplasm [40, 41], corroborating our identification of osmophores based on the massive amounts of these structures in glandular trichomes. The restricted chamber-like environment close to the trichomes may hold the highest concentration of VOCs, and their diffusion across the cuniculus to the atmosphere may act as a clue for pollinators. In this sense, the cuniculus may act as a VOC holder, in the same sense as the nectar holders (i.e., where nectar is confined before being released) found in Asclepiadaceae flowers [42]. Thus, the cuniculus may provide tactile and olfactory stimuli to pollinators, enhancing their attraction and exploratory behavior, as suggested in other orchid groups [17, 43].

## Conclusions

The recurrent and apparently irreversible pattern of nectar loss in *Epidendrum* suggests that food deception may constitute an alternative evolutionarily stable strategy, as observed in other orchid groups [17, 18]. The evolution of rewardless species may have several biological implications, such as altering pollinator behavior, patterns of gene exchange, type of reproductive isolation, and rates of speciation. The presence and absence of nectar in different *Epidendrum* species offer an opportunity to explore the ecological forces associated to the evolution of reward and rewardless species. Unfortunately, *Epidendrum* is one of the orchid genus poorly understood, despite its impressive diversity. Information about pollinators, reproductive biology and seed set in reward and rewardless species are needed, in order to understand the ecological conditions in which both strategies evolve. Population genetic and phylogeographic studies have focused mainly species from group *Amphyglottium* (reviewed by [9]) which is composed mainly by nectarless species, reducing our ability to understand the patterns of gene exchange in nectar offering species. For example, our hypothesis that ornamented nectaries may provide tactile stimuli to pollinators need to be investigated by monitoring pollinators behavior, observing the depth of penetration, period of residence and movement of the proboscis within the cuniculus. We are aware that, giving the size of the genus, our sample is limited, and some conclusions may be interpreted cautiously, such as the inference of nectar secreting species using the inner morphology of the cuniculus. Nonetheless, our study offer new insights and hypothesis that need to be tested in future studies, in order to clarify what evolutionary mechanisms are involved in the diversification of hyper diverse genera such as *Epidendrum*.

## Methods

### Study system and taxon sampling

The genus *Epidendrum* has been the focus of major taxonomic treatments in the last few years, focused mainly on describing new species. We focused our analysis on the group *Amphyglottium* due to its monophyly [21] and the availability of fresh flowers for anatomical analyses. Flowers were sampled from 27 species (31 accessions) maintained in the living orchid collection at the Instituto de Botanica (São Paulo, Brazil), from which 20 species (24 accessions) were also included in the phylogeny. The phylogenetic inference was based on sequence data obtained from GenBank and new data generated in the present study. Details are given in Additional file 4: Table S1.

### Anatomical analyses

The cuniculus glands from the flowers of 27 selected species (see Additional file 4: Table S1) were investigated at their secretory stage, when the flowers were at anthesis. The presence of nectar was assessed and established by glucose strips (whenever possible) and in situ by histochemical detection of reducing sugars.

Ovaries were excised from flowers, longitudinally sectioned to observe the epidermis of the cuniculus, and immediately fixed in 2.5% glutaraldehyde in phosphate buffer (pH 7.4, 0.1 M) for 24 h. The samples were washed in phosphate buffer, dehydrated in an ethanol series, and stored in 70% ethanol. The material was further dehydrated to 100% ethanol and rinsed in a hexamethyldisilazane (HMDS) series (33.3, 50.0, and 66.6% *v*/v in 100% ethanol) and then three times in 100% HMDS for 1 min each [44] to dry the material. Samples were mounted on stubs, coated with gold in a Leica ACE 200 sputtering system and viewed with a FEI Quanta 250 at 10 kV. Digital images were edited using Adobe Photoshop version 7.0 (Adobe Systems, Inc., San Jose, California, USA).

Fixed ovaries (see above) were embedded using standard methods for Leica historesin (Heraeus Kulzer, Germany) and serially sectioned at 5-μm thicknesses. The sections were stained with toluidine blue O [45] and mounted in water. Observations and digital images were acquired with an Olympus BX53 compound microscope equipped with an Olympus Q-Color 5 digital camera and Image Pro Express 6.3 software.

The presence of glucose in the exudate was assessed with a glucose strip test (Inlab Diagnostica; Alamar Tecno Cientifica, Buenos Aires, Argentina) whenever the nectar was profuse enough to be tested. Immediately after the excision of ovaries from fresh flowers, their internal liquid content was removed with microcapillary tubes and applied to glucose strips. For the in situ detection of reducing sugars, fresh cross-sectioned ovaries were treated with Fehling’s reagent, equal parts copper (II) sulfate 6.93% w:v, sodium potassium tartrate 34.6, and 12% sodium hydroxide w:w:v solutions heated to a pre-boiling temperature. After treatment, the sections were immediately observed under a light microscope.

Ovarian regions were isolated and immediately fixed at room temperature in 1.5% glutaraldehyde and 0.2 M cacodylate buffer, pH 7.25. After several washes with cacodylate buffer, the material was post-fixed in 1% OsO_4_ for 2 h, dehydrated to 100% ethanol, and them embedded in LR White embedding medium (Ted Pella Inc., Redding, CA, USA). The sections were mounted on copper grids and viewed with a Jeol JEM 2100 TEM at 80 kV.

### Molecular methods

For molecular analysis, leaf samples were sliced into small pieces and transferred to silica gel for drying. Total genomic DNA was extracted as described by Pinheiro et al. [21]. We PCR-amplified and sequenced five plastid regions (matK, rbcL, trnL–trnF, trnT–trnL, and rpl32–trnL) and one nuclear region, the internal transcribed spacer (ITS) region (including the 5.8S gene), using the protocols and primers from Pinheiro et al. [46] for trnL–trnF, trnT–trnL, and rpl32–trnL, and van den Berg et al. [47] for matK, rbcL, and ITS. We believe more nuclear markers would be helpful but, due to the extensive chromosome variation found in *Epidendrum* species, it is very difficult to amplify and sequence low copy nuclear regions.

Amplification products were sequenced at Macrogen, Inc. (Seoul, South Korea) using the above PCR primers. Sequences were edited and aligned using the MUSCLE algorithm with default settings implemented in GENEIOUS version 5.4 software (Biomatters, Ltd., Auckland, New Zealand). We also performed a final editing of the sequence alignment by hand. For all plastid and nuclear regions, the sequence length variation among species was low. Hence, gaps were generally treated as missing data. GenBank sequence accession numbers and voucher details are summarized in Additional file 4: Table S1. We included outgroups and multiple intra-specific samples in the phylogenetic analyses, resulting in a total of 58 terminal accessions from 54 species. Two misidentifications in Pinheiro et al. [21] were corrected: the specimen identified as *E. myrmecophorum* was re-identified as *E. orchidiflorum*, and the specimen identified as *E. incisum* was re-identified as *E. macrocarpum*.

### Phylogeny and time calibration of *Epidendrum* divergence

The joint posterior distribution of topologies and divergence times was estimated using Bayesian Markov chain Monte Carlo (MCMC) implemented in BEAST 1.8.1 software [48] in a two-step analysis with different calibration schemes. First, we estimated the divergence time of the main Orchidaceae clades with the following node calibrations: age for subtribe Goodyerinae, 15–20 Ma based on fossil records of orchid pollinia [49]; and divergence time estimates [50] for subfamily Epidendroideae (mean: 49 Ma), and the informal group “Higher Epidendroids” (mean: 39 Ma). This data set included the plastid DNA sequences ribulose-bisphosphate carboxylase (rbcL) and maturase K (matK) for 39 orchids from the aligned sequence matrix by Gustafsson et al. [50], plus ten *Epidendrum* species, resulting in 49 orchid taxa.

The second-step analysis was performed for *Epidendrum* using the age estimates of the first analysis as a secondary calibration: 1) age for subtribe Laellinae, normal prior distribution with mean 20.1 Ma and standard deviation 3.8 (95% CI, 13.85–26.35 Ma); and 2) age for clade “*Meiracyllium*, *Cattleya* and *Epidendrum*”: normal prior distribution with mean 12.62 Ma and standard deviation 4.2 (95% CI, 5.72–19.53 Ma). Fifteen species (19 accessions) from group *Amphyglottium* were analyzed, and 32 *Epidendrum* species were used as outgroups, plus 7 species belonging to four different Laeliinae genera (*Arpophyllum*, *Cattleya*, *Encyclia*, and *Meiracyllium*). This data set consisted of five plastid regions (matK, rbcL, trn–trnF, trnT–trnL, and rpl32–trnL) and one nuclear region, the ITS region (including the 5.8S gene). We also included the same rbcL and matK sequences used for the outgroups and the *Epidendrum* species used in the first-step calibration analysis. The General Time Reversible (GTR) model was selected for the nuclear marker ITS and the plastid markers rpl32–trnL, trnL–trnF, and trnT–trnL. The GTR + Γ + I model was selected for plastid markers matK and rbcL. All partitions were analyzed linked with an uncorrelated lognormal relaxed clock prior and a Yule tree prior. Three separated runs were performed in BEAST with 50,000,000 generations and a sampling frequency of every 1000 generations. Log files were analyzed with Tracer v. 1.6 [51] to assess convergence and determine whether the effective sample sizes were larger than 200 for all parameters. The resulting trees were then combined with LogCombiner v. 1.8.1, with a burn-in of 10%. A maximum credibility tree was produced using TreeAnnotator v. 1.8.1 [52].

Preliminary analysis in our data revealed a covariance with a mean of 0.12 and 95% confidence intervals ranging from 0.0551–0.3055 (values close to zero indicate low covariance). The low covariance found in our data favor the use of BEAST instead of R8S [53]. As reported in the literature [50, 54], using the penalized likelihood with uncorrelated data would fail to obtain robust age estimates. Thus, it would be difficult to compare age estimates obtained by R8S and Beast, precluding categorical assertion son which of these methods yields the most correct divergence estimate [55].

The congruence between nuclear and plastid data sets was assessed by comparing the topologies and posterior probabilities of the strict consensus trees of two main data partitions, the nuclear ITS, and all plastid regions together, according to the method of Xiang et al. [56]. The test was performed by visually comparing the support and resolution of each of the clades in the separate analyses that had a higher posterior probability (PP) > 90 [57]. Because most incongruences were restricted to clades showing low support values (Bayesian posterior probability [BPP] < 0.90 – data not shown), only the Bayesian inference based on the combined nuclear and plastid data sets was used for phylogenetic inference, divergence time estimations, and analysis of character evolution.

### Evolution of nectar gain and loss

Nectar presence and absence was directly inferred from anatomical observations and histochemical tests. In addition, we interpreted the presence of an ornamented cuniculus as a sign of nectar absence. Conversely, the presence of an unornamented cuniculus was indicative of nectar presence, according to our results (Additional file 4: Table S1). Ancestral character state reconstruction analysis was used to map the transitions between nectar absence (NA) and nectar presence (NP) in *Epidendrum* species. The NA and NP states were coded as binary data (0 and 1, respectively) and optimized onto the combined (total evidence = nuclear + plastid datasets) Bayesian inference tree under a Maximum Likelihood criterion using the Mesquite v. 3.31 package [58]. The number and directionality of transitions between NA and NP states were quantified using the Summarize State Changes Over Trees function in Mesquite. The number of state transitions was summarized over 10,000 chronograms.

Differences in the rates of transitions between NA and NP were tested following Pinheiro et al. [59], using 10,000 chronograms. Three models were compared using likelihood ratio tests: (1) the unrestricted model, in which the probability of the two types of transitions, from NA to NP (q_01_) and the converse (q_10_), were calculated; (2) a restricted model in which only NA to NP transitions were permitted (i.e., q_10_ = 0); and (3) an alternative, restricted model in which only NP to NA transitions were permitted (i.e., q_01_ = 0). The likelihood ratio test was used to compare the two likelihoods derived from the unrestricted and each of the restricted models. In the restricted models, ancestral states were fixed to NA or NP. MCMC analyses were performed using the BayesMultistates program [60] implemented in BayesTraits v. 3.0.1 (http://www.evolution.rdg.ac.uk/BayesTraits.html). The MCMC analyses were run for 5,050,000 generations, with a uniform prior distribution and a burn-in of 50,000 generations.

The presence and absence of nectar was directly inferred for the 27 species investigated in detail based on anatomical observations. We also used an indirect approach to infer nectar presence/absence in species for which fresh material was not available, using associations between epidermal characters and the presence/absence of nectar. We found a strong association between an ornamented cuniculus (presence of epidermal unicellular trichomes) and lack of nectar. In contrast, an unornamented cuniculus (uniform epidermis lacking trichomes) was always associated with nectar presence (see description in results). Thus, to estimate the proportion of nectarless *Epidendrum* species, we used the species described by Hágsater et al. in *Icones Orchidacearum* (Additional file 3: Table S2) as a proxy for the whole genus. We restricted our analysis to the species described in *Icones Orchidacearum* due to the detailed and standardized nature of its flower descriptions, providing information for almost the entire genus (approximately 1100 species). Detailed distribution maps for all species analyzed here are also provided by *Icones Orchidacearum*.

## Additional files

Additional file 1: **Figure S1.** Details of *Epidendrum* flowers, showing the cuniculi. (A) *E*. *coronatum* flower*.* The red rectangle indicates the pedicel region dissected by longitudinal sections in the remaining pictures (B–D). (B) Detail of the cuniculus of *E. cristatum.* The dotted line indicates the area from which most samples were taken for anatomical analyses. (C) Unornamented cuniculus of *E. orchidiflorum*. (D) Ornamented cuniculus of *E. fulgens.* Scale bars = 1.0 cm. (PDF 6036 kb)

Additional file 2: **Figure S2.** Time-calibrated tree of the Orchidaceae, focusing on the subfamilies Orchidoideae and Epidendroideae, based on matK and rbcL plastid regions, estimated using BEAST software. Thin branches indicate posterior probabilities below 0.9. Circles indicate age-constrained nodes. The yellow circle indicates the calibration point for subtribe Goodyerinae (*Pachyplectron–Dossinia*), the blue circle indicates the calibration point for Epidendroideae, and the green circle indicates the calibration point for the Higher Epidendroids. Numbers at nodes represent median ages in millions of years (Ma). (PDF 665 kb)

Additional file 3: **Table S2.** Species described in Icones Orchidacearum (1993–2016) showing ornamented cuniculus, including their informal group and specific reference. (DOCX 20 kb)

Additional file 4: **Table S1.** *Epidendrum* species and allied genera analyzed in this study, including GenBank access numbers by molecular marker, including the type of analyses performed (PC = primary calibration, SC = secondary calibration, AN = anatomy), and presence or absence of nectar with the corresponding source of information. (DOCX 30 kb)

## Abbreviations

AFLP: Amplified fragment length polymorphism
BPP: Bayesian posterior probability
GTR: General Time Reversible model
HMDS: Hexamethyldisilazane
ITS: Internal transcribed spacer
Ma: Million of years
MCMC: Markov-chain Monte Carlo
NA: Nectar absence
NP: Nectar presence
PP: Posterior probability
SEM: Scanning electron microscopy
TEM: Transmission electron microscopy
VOC: Volatile organic compounds
