# Supplementary figures and images for: Unidirectional transitions in nectar gain and loss suggest food deception is a stable evolutionary strategy in Epidendrum (Orchidaceae): insights from anatomical and molecular evidence
Source: BMC Plant Biol. 2018 Sep 4;18:179. doi: 10.1186/s12870-018-1398-y (PMC6122447; doi:10.1186/s12870-018-1398-y)

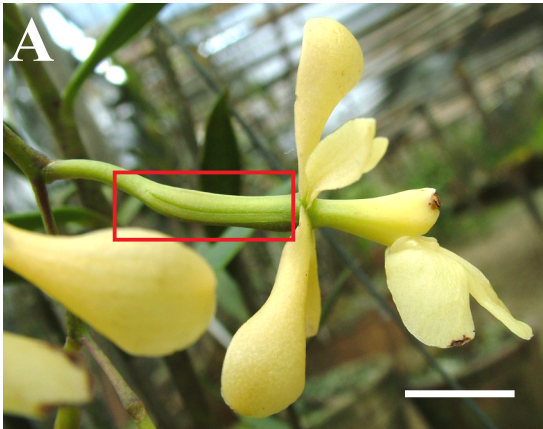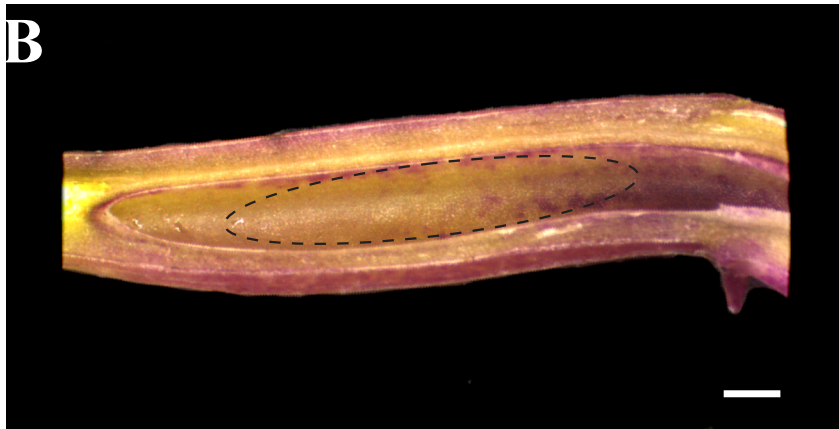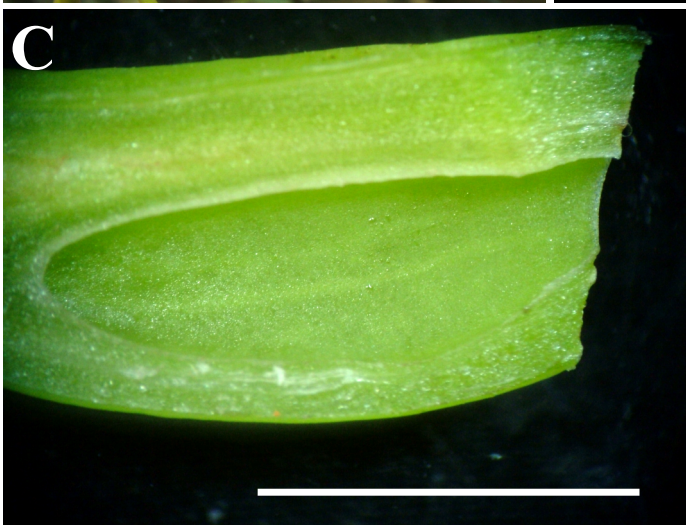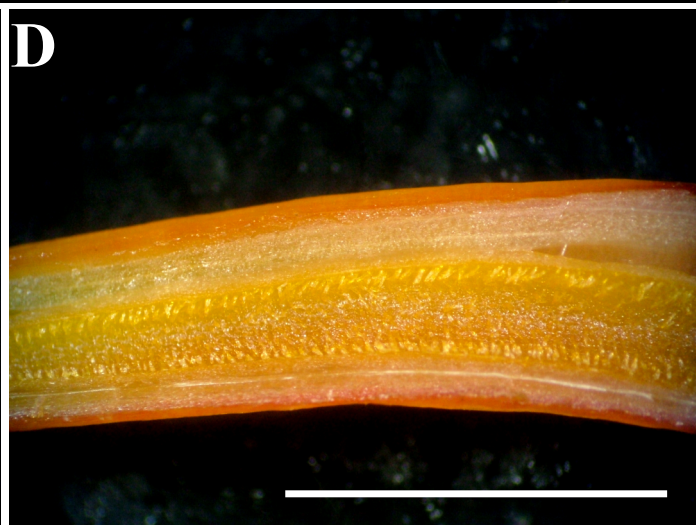

Supplement: Supplementary file 1 — Figure S1. Details of Epidendrum flowers, showing the cuniculi. (A) E. coronatum flower. The red rectangle indicates the pedicel region dissected by longitudinal sections in the remaining pictures (B–D). (B) Detail of the cuniculus of E. cristatum. The dotted line indicates the area from which most samples were taken for anatomical analyses. (C) Unornamented cuniculus of E. orchidiflorum. (D) Ornamented cuniculus of E. fulgens. Scale bars = 1.0 cm. (PDF 6036 kb) [file 12870_2018_1398_MOESM1_ESM.pdf]

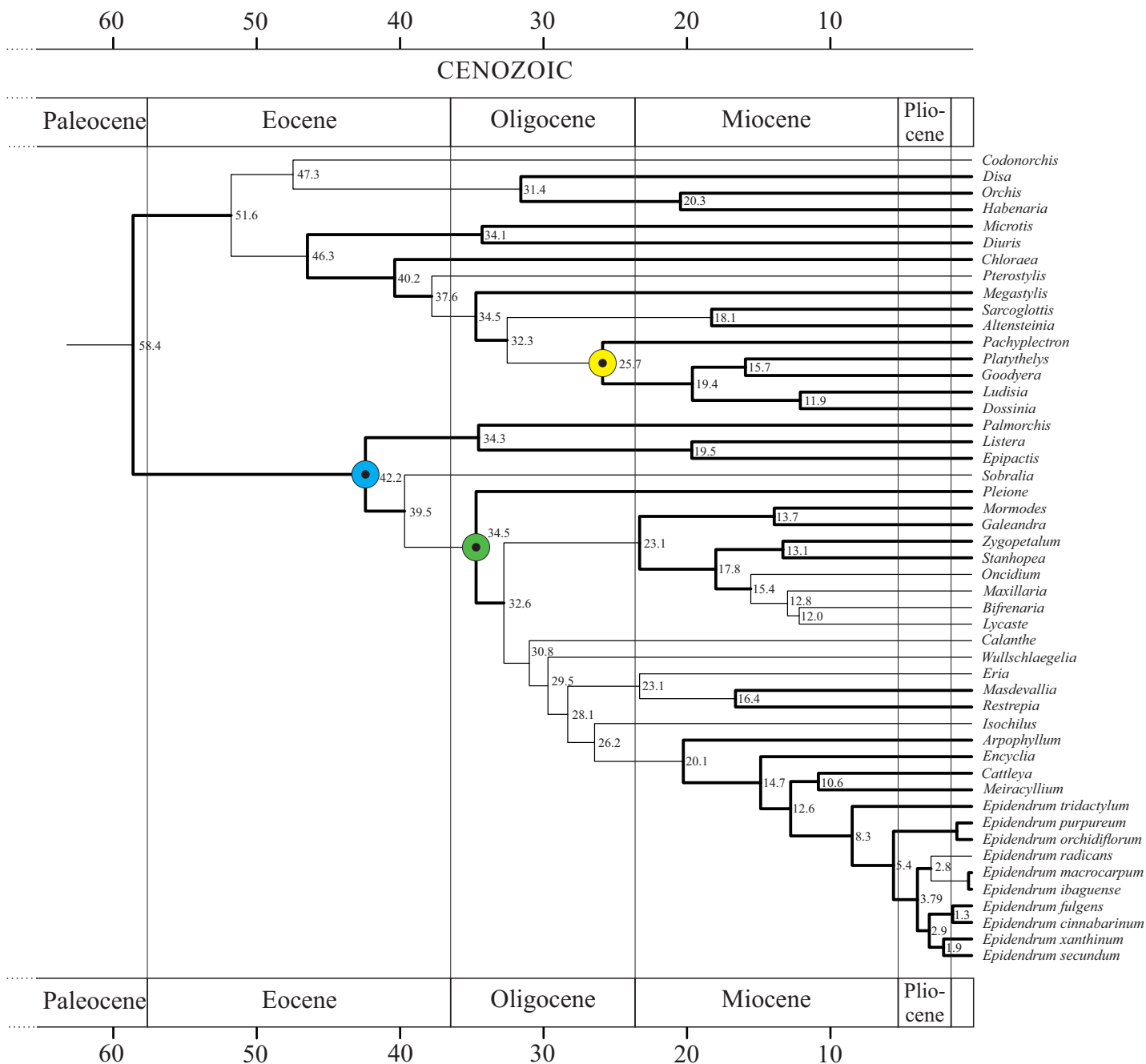

Supplement: Supplementary file 2 — Figure S2. Time-calibrated tree of the Orchidaceae, focusing on the subfamilies Orchidoideae and Epidendroideae, based on matK and rbcL plastid regions, estimated using BEAST software. Thin branches indicate posterior probabilities below 0.9. Circles indicate age-constrained nodes. The yellow circle indicates the calibration point for subtribe Goodyerinae (Pachyplectron–Dossinia), the blue circle indicates the calibration point for Epidendroideae, and the green circle indicates the calibration point for the Higher Epidendroids. Numbers at nodes represent median ages in millions of years (Ma). (PDF 665 kb) [file 12870_2018_1398_MOESM2_ESM.pdf]
