# Supplementary material for: Unidirectional transitions in nectar gain and loss suggest food deception is a stable evolutionary strategy in Epidendrum (Orchidaceae): insights from anatomical and molecular evidence
Source: BMC Plant Biol. 2018 Sep 4;18:179. doi: 10.1186/s12870-018-1398-y (PMC6122447; doi:10.1186/s12870-018-1398-y)
Supplement: Supplementary file 3 — Table S2. Species described in Icones Orchidacearum (1993–2016) showing ornamented cuniculus, including their informal group and specific reference. (DOCX 20 kb) [file 12870_2018_1398_MOESM3_ESM.docx]

Table S2. Species described in Icones Orchidacearum (1993-2016) showing ornamented cuniculus, including their informal group and specific reference.

| Species | Informal group | Reference |
| --- | --- | --- |
| *E. ackermanii* | Amphiglottium | Hágsater & Sánchez-Saldana 2004 |
| *E. acroscopeum* | Amphiglottium | Hágsater & Sánchez-Saldana 2006 |
| *E. aura-usecheae* | Amphiglottium | Hágsater & Sánchez-Saldana 2013 |
| *E. bangii* | Macrostachyum | Hágsater & Sánchez-Saldana 2015 |
| *E. baumannianum* | Amphiglottium | Hágsater & Sánchez-Saldana 2008 |
| *E. bicuniculatum* | Noewilliansia | Hágsater & Sánchez-Saldana 2007 |
| *E. borealistachyum* | Macrostachyum | Hágsater & Sánchez-Saldana 2015 |
| *E. brachycorymbsum* | Schistochilum | Hágsater & Sánchez-Saldana 2004 |
| *E. brachyoothistachyum* | Macrostachyum | Hágsater & Sánchez-Saldana 2015 |
| *E. brachystelestachyum* | Macrostachyum | Hágsater & Sánchez-Saldana 2015 |
| *E. bractiacuminatum* | Macrostachyum | Hágsater, Sánchez-Saldana & García-Cruz 1999 |
| *E. bryophilum* | Elleanthoides/Macrostachyum? | Hágsater & Sánchez-Saldana 2001 |
| *E. campylorhachis* | Macrostachyum | Hágsater & Sánchez-Saldana 2004 |
| *E. campylostele* | Elleanthoides/Macrostachyum? | Hágsater & Sánchez-Saldana 2004 |
| *E. cardiostachyum* | Macrostachyum | Hágsater & Sánchez-Saldana 2015 |
| *E. chiguindense* | Carolii | Hágsater & Sánchez-Saldana 2004 |
| *E. chilcalorum* | Pilcuense/Macrostachyum? | Hágsater & Sánchez-Saldana 2015 |
| *E. chrysanthum* | Elleanthoides/Macrostachyum? | Hágsater & Sánchez-Saldana 2001 |
| *E. coordinatum* | Macrostachyum | Hágsater & Sánchez-Saldana 2015 |
| *E. coroicoense* | Amphiglottium | Hágsater & Sánchez-Saldana 2015 |
| *E. crescentilobum* | Oerstedella | Hágsater & Sánchez-Saldana 2006 |
| *E. dactyloclinum* | Physinga | Hágsater & Sánchez-Saldana 2001 |
| *E. deltastachyum* | Macrostachyum | Hágsater & Sánchez-Saldana 2015 |
| *E. dermatanthum* | Macrostachyum | Hágsater & Sánchez-Saldana 2015 |
| *E. ellanthodiceras* | Elleanthoides/Macrostachyum? | Hágsater & Sánchez-Saldana 2004 |
| *E. ellanthoides* | Elleanthoides/Macrostachyum? | Hágsater & Sánchez-Saldana 2006 |
| *E. erosum* | Scabrum | Hágsater & Sánchez-Saldana 2007 |
| *E. flexuosum* | Amphiglottium | Hágsater & Sánchez-Saldana 2008 |
| *E. franckei* | Amphiglottium | Hágsater & Sánchez-Saldana 2004 |
| *E. fritzicardium* | Andean | Hágsater & Sánchez-Saldana 2009 |
| *E. fritzimegalotylosum* | Andean | Hágsater & Sánchez-Saldana 2009 |
| *E. gerlachianum* | Andean | Hágsater & Sánchez-Saldana 2013 |
| *E. glossoclinium* | Albertii | Hágsater & Sánchez-Saldana 2007 |
| *E. golondrinense* | Macrostachyum/Aquaticum? | Hágsater & Sánchez-Saldana 2001 |
| *E. grammipetalostachyum* | Macrostachyum | Hágsater & Sánchez-Saldana 2013 |
| *E. grayi* | Macrostachyum/Aquaticum? | Hágsater 1993 |
| *E. guaramacalense* | Elleanthoides/Macrostachyum? | Hágsater & Sánchez-Saldana 2004 |
| *E. hawkesii* | Amphiglottium | Hágsater & Sánchez-Saldana 2008 |
| *E. herperium* | Elleanthoides/Macrostachyum? | Hágsater & Sánchez-Saldana 2004 |
| *E. holmnielsenii* | Arbuscula | Hágsater & Sánchez-Saldana 2001 |
| *E. humeadorense* | Macrostachyum | Hágsater, Sánchez-Saldana & García-Cruz 1999 |
| *E. igneum* | Amphiglottium | Hágsater & Sánchez-Saldana 2004 |
| *E. imperator* | Amphiglottium | Hágsater & Sánchez-Saldana 2004 |
| *E. indanzense* | Anceps | Hágsater 1993 |
| *E. insignificans* | Macrostachyum/Aquaticum? | Hágsater 1993 |
| *E. ionostachyum* | Macrostachyum | Hágsater & Sánchez-Saldana 2016 |
| *E. laurelense* | Amphiglottium | Hágsater & Sánchez-Saldana 2001 |
| *E. lembotylosum* | Elleanthoides/Macrostachyum? | Hágsater & Sánchez-Saldana 2004 |
| *E. leucolistron* | Elleanthoides/Macrostachyum? | Hágsater & Sánchez-Saldana 2013 |
| *E. lima* | Andean | Hágsater & Sánchez-Saldana 2009 |
| *E. lindae* | Macrostachyum/Aquaticum? | Hágsater, Sánchez-Saldana & García-Cruz 1999 |
| *E. lindae* | Macrostachyum/Aquaticum? | Hágsater & Sánchez-Saldana 2010 |
| *E. lindamazonicum* | Macrostachyum/Aquaticum? | Hágsater & Sánchez-Saldana 2010 |
| *E. macphersonii* | Macrostachyum | Hágsater & Sánchez-Saldana 2015 |
| *E. macrostachyum* | Macrostachyum | Hágsater & Sánchez-Saldana 2013 |
| *E. magnibracteum* | Macrostachyum | Hágsater & Sánchez-Saldana 2015 |
| *E. microcarpum* | Macrostachyum | Hágsater & Sánchez-Saldana 2001 |
| *E. microrigidiflorum* | Macrostachyum | Hágsater & Sánchez-Saldana 2006 |
| *E. molaui* | Cardiophyllum | Hágsater & Sánchez-Saldana 2001 |
| *E. momipsis* | Amphiglottium | Hágsater & Sánchez-Saldana 2004 |
| *E. montispichinchense* | Elleanthoides/Macrostachyum? | Hágsater & Sánchez-Saldana 2007 |
| *E. munchiquense* | Macrostachyum | Hágsater & Sánchez-Saldana 2015 |
| *E. neudeckei* | Piperium | Hágsater & Sánchez-Saldana 2010 |
| *E. obovatipetalum* | Macrostachyum | Hágsater 1993 |
| *E. odontopetalum* | Amphiglottium | Hágsater & Sánchez-Saldana 2008 |
| *E. odontostachyum* | Macrostachyum | Hágsater & Sánchez-Saldana 2015 |
| *E. oripicoranense* | Elleanthoides/Macrostachyum? | Hágsater & Sánchez-Saldana 2010 |
| *E. orthophyllum* | Macrostachyum | Hágsater 1993 |
| *E. oxycalix* | Macrostachyum | Hágsater 1993 |
| *E. palaciosii* | Macrostachyum | Hágsater 1993 |
| *E. parkisonianum* | Falcatum | Hágsater & Sánchez-Saldana 2010 |
| *E. peraltum* | Pseudoepidendrum | Hágsater & Sánchez-Saldana 2010 |
| *E. phillowerckelei* | Neowilliansia | Hágsater & Sánchez-Saldana 2006 |
| *E. piconeblinaense* | Elleanthoides/Macrostachyum? | Hágsater & Sánchez-Saldana 2004 |
| *E. pilcuense* | Macrostachyum/Aquaticum? | Hágsater 1993 |
| *E. pittieri* | Andean | Hágsater & Sánchez-Saldana 2009 |
| *E. platypetalum* | Megalospathum | Hágsater & Sánchez-Saldana 2001 |
| *E. platystachyum* | Macrostachyum | Hágsater & Sánchez-Saldana 2004 |
| *E. polyanthostachyum* | Macrostachyum | Hágsater & Sánchez-Saldana 2013 |
| *E. porphyrostachyum* | Macrostachyum | Hágsater & Sánchez-Saldana 2015 |
| *E. portokalium* | Amphiglottium | Hágsater & Sánchez-Saldana 2004 |
| *E. probosantherum* | Macrostachyum | Hágsater & Sánchez-Saldana 2010 |
| *E. pseudogramineum* | Macrostachyum | Hágsater & Sánchez-Saldana 2006 |
| *E. ptochium* | Amphiglottium | Hágsater & Sánchez-Saldana 2006 |
| *E. puracestachyum* | Macrostachyum | Hágsater & Sánchez-Saldana 2015 |
| *E. rauhii* | Amphiglottium | Hágsater & Sánchez-Saldana 2004 |
| *E. revertianum* | Amphiglottium | Hágsater & Sánchez-Saldana 2008 |
| *E. rhodanthum* | Macrostachyum/Aquaticum? | Hágsater, Sánchez-Saldana & García-Cruz 1999 |
| *E. rhombobrachyphyllum* | Elleanthoides/Macrostachyum? | Hágsater & Sánchez-Saldana 2013 |
| *E. rigidiflorum* | Macrostachyum | Hágsater & Sánchez-Saldana 2008 |
| *E. rostrigerum* | Macrostachyum | Hágsater & Sánchez-Saldana 2015 |
| *E. rubioi* | Macrostachyum/Aquaticum? | Hágsater 1993 |
| *E. rugulosum* | Macrostachyum/aquaticum? | Hágsater & Sánchez-Saldana 2015 |
| *E. saccatum* | Elleanthoides/Macrostachyum? | Hágsater & Sánchez-Saldana 2001 |
| *E. sarcostachyum* | Macrostachyum | Hágsater & Sánchez-Saldana 2015 |
| *E. sigmostachyum* | Macrostachyum | Hágsater & Sánchez-Saldana 2015 |
| *E. silverstonei* | Scabrum | Hágsater, Sánchez-Saldana & García-Cruz 1999 |
| *E. spasmosum* | Elleanthoides/Macrostachyum? | Hágsater & Sánchez-Saldana 2004 |
| *E. sphenostele* | Neowilliansia | Hágsater & Sánchez-Saldana 2007 |
| *E. stenobractistachyum* | Macrostachyum | Hágsater & Sánchez-Saldana 2015 |
| *E. stenocalymmum* | Macrostachyum/aquaticum? | Hágsater & Sánchez-Saldana 2004 |
| *E. sucumbiense* | Pseudoepidendrum | Hágsater & Sánchez-Saldana 2001 |
| *E. sumacostachyum* | Macrostachyum | Hágsater & Sánchez-Saldana 2015 |
| *E. susannae* | Amphiglottium | Hágsater & Sánchez-Saldana 2013 |
| *E. thermophilum* | Macrostachyum/Aquaticum? | Hágsater 1993 |
| *E. thompsonii* | Cardiophyllum | Hágsater, Sánchez-Saldana & García-Cruz 1999 |
| *E. tolimense* | Elleanthoides/Macrostachyum? | Hágsater & Sánchez-Saldana 2008 |
| *E. torraense* | Macrostachyum | Hágsater & Sánchez-Saldana 2001 |
| *E. trullichilum* | Macrostachyum | Hágsater, Sánchez-Saldana & García-Cruz 1999 |
| *E. tulcanense* | Amphiglottium | Hágsater & Sánchez-Saldana 2013 |
| *E. vallisoletanum* | Pseudoepidendrum | Hágsater & Sánchez-Saldana 2013 |
| *E. wercklei* | Neowilliansia | Hágsater & Sánchez-Saldana 2007 |
| *E. witherspooniorum* | Macrostachyum/aquaticum? | Hágsater & Sánchez-Saldana 2004 |

**Literature Cited in Supplementary Table S2**

**Hágsater E. 1993.** *Icones Orchidacearum: fascicle 2*. México: Herbario AMO.

**Hágsater E, Sánchez-Saldana LM. 2007.** *Icones Orchidacearum: fascicle 9*. México: Herbario AMO.

**Hágsater E, Sánchez-Saldana LM.** **2008.** *Icones Orchidacearum: fascicle 11*. México: Herbario AMO.

**Hágsater E, Sánchez-Saldana LM.** **2001.** Icones Orchidacearum: fascicle 4. México: Herbario AMO.

**Hágsater E, Sánchez-Saldana LM.** **2006.** *Icones Orchidacearum: fascicle 8*. México: Herbario AMO.

**Hágsater E, Sánchez-Saldana LM.** **2009.** *Icones Orchidacearum: fascicle 12*. México: Herbario AMO.

**Hágsater E, Sánchez-Saldana LM. 2010.** *Icones Orchidacearum: fascicle 13*. México: Herbario AMO.

**Hágsater E, Sánchez-Saldana LM, García-Cruz CJ. 1999.** *Icones Orchidacearum: fascicle 3*. México: Herbario AMO.

**Hágsater E, Sánchez-Saldana LM. 2004.** *Icones Orchidacearum: fascicle 7*. México: Herbario AMO.

**Hágsater E, Sánchez-Saldana LM. 2013.** *Icones Orchidacearum: fascicle 14*. México: Herbario AMO.

**Hágsater E, Sánchez-Saldana LM. 2015.** *Icones Orchidacearum: fascicle 15(1).* México: Herbario AMO.

**Hágsater E, Sánchez-Saldana LM. 2016.** *Icones Orchidacearum: fascicle 15(2)*. México: Herbario AMO.
