# Supplementary material for: Unidirectional transitions in nectar gain and loss suggest food deception is a stable evolutionary strategy in Epidendrum (Orchidaceae): insights from anatomical and molecular evidence
Source: BMC Plant Biol. 2018 Sep 4;18:179. doi: 10.1186/s12870-018-1398-y (PMC6122447; doi:10.1186/s12870-018-1398-y)
Supplement: Supplementary file 4 — Table S1. Epidendrum species and allied genera analyzed in this study, including GenBank access numbers by molecular marker, including the type of analyses performed (PC = primary calibration, SC = secondary calibration, AN = anatomy), and presence or absence of nectar with the corresponding source of information. (DOCX 30 kb) [file 12870_2018_1398_MOESM4_ESM.docx]

TABLE S1. *Epidendrum* species and allied genera analyzed in this study, including GenBank access numbers by molecular marker, including the type of analyses performed (PC = primary calibration, SC = secondary calibration, AN = anatomy), and presence or absence of nectar with the corresponding source of information. GenBank access numbers in bold correspond to sequences generated in this study.

| Group/Genus | Species (Voucher) | Analysis | Inference of nectar presence | GenBank access number | | | | | |
| --- | --- | --- | --- | --- | --- | --- | --- | --- | --- |
|  |  |  |  | matK | rbcL | trnL–F | trnT–L | rpl32–trnL | ITS |
| Group *Amphyglottium* | *Epidendrum calanthum* (F. Pinheiro 606 - SP) | SC, AN | Absent (this study) | - | - | JQ646008 | JQ645988 | JQ645968 | **MH218753** |
|  | *E. cinnabarinum* (F. Pinheiro 609 - SP) | PC, SC, AN | Absent (this study) | **MH218774** | **MH218787** | JQ646011 | JQ645991 | JQ645971 | **MH218760** |
|  | *E. cochlidium* (F Pinheiro 556 - SP) | SC, AN | Absent (this study) | - | - | FJ869423 | **MH218803** | **MH218797** | **MH218749** |
|  | *E. denticulatum* (F. Pinheiro 610 - SP) | SC, AN | Absent (this study) | - | - | JQ646012 | JQ645992 | JQ645972 | **MH218754** |
|  | *E. flammeus* (F. Pinheiro 612 - SP) | SC, AN | Absent (this study) | - | - | JQ646014 | JQ645994 | JQ645974 | **MH218763** |
|  | *E. flexuosum* (F. Pinheiro 575 - SP) | SC, AN | Absent (this study) | - | - | FJ869421 | **MH218807** | **MH218798** | **MH218761** |
|  | *E. fulgens* (F. Pinheiro 631 - SP) | PC, SC, AN | Absent (this study) | **MH218776** | **MH218786** | JQ646018 | JQ645998 | JQ645978 | **MH218755** |
|  | *E. ibaguense* (F. Pinheiro 555 - SP) | PC, SC, AN | Absent (this study) | **MH218772** | **MH218791** | FJ869399 | **MH218806** | **MH218795** | **MH218758** |
|  | *E. macrocarpum* (F. Pinheiro 532 - SP) | PC, SC, AN | Absent (this study) | **MH218773** | **MH218792** | FJ869400 | - | **MH218794** | **MH218759** |
|  | *E. macrocarpum* (F. Pinheiro 618 - SP) | SC, AN | Absent (this study) | - | - | JQ646020 | JQ646000 | JQ645980 | **MH218762** |
|  | *E. orchidiflorum* (F. Pinheiro 619 - SP) | PC, SC, AN | Absent (this study) | **MH218764** | **MH218783** | JQ646021 | JQ646001 | JQ645981 | **MH218756** |
|  | *E. puniceoluteum* (F. Pinheiro 621 - SP) | SC, AN | Absent (this study) | **MH218775** | - | JQ646023 | JQ646003 | JQ645983 | **MH218752** |
|  | *E. purpureum* (F. Pinheiro 530 - SP) | PC, SC, AN | Absent (this study) | **MH218765** | **MH218784** | FJ869403 | **MH218804** | **MH218793** | **MH218757** |
|  | *E. radicans* (F. Pinheiro 623 - SP) | PC, SC, AN | Absent (this study) | **MH218771** | **MH218788** | JQ646025 | JQ646005 | JQ645985 | **-** |
|  | *E. secundum* (F Pinheiro 624 - SP) | PC, SC, AN | Absent (this study) | **MH218767** | **MH218781** | JQ646026 | JQ646006 | JQ645986 | **MH218750** |
|  | *E. secundum* (F Pinheiro 561 - SP) | SC, AN | Absent (this study) | **MH218768** | **MH218782** | FJ869419 | - | - | **MH218751** |
|  | *E. xanthinum* 1 (F. Pinheiro 625 - SP) | PC, SC, AN | Absent (this study) | **MH218769** | **MH218789** | JQ646027 | JQ646007 | JQ645987 | **MH218746** |
|  | *E. xanthinum* 2 (F. Pinheiro 570 - SP) | SC, AN | Absent (this study) | **MH218770** | **MH218790** | FJ869415 | **MH218805** | **MH218796** | **MH218747** |
|  | *E. xanthinum* 3 (F. Pinheiro 546 - SP) | SC, AN | Absent (this study) | **MH218766** | **MH218785** | FJ869416 | - | - | **MH218748** |
| *Epidendrum* outgroups | *E. alfaroi* (DB 1641) | SC | Present (Hágsater & Sánchez-Saldana 2007) | EU214344.1 | - | - | - | - | - |
|  | *E. anceps* (F. Pinheiro 644 - SP) | SC, AN | Absent (this study) | - | - | **MH218801** | - | - | **MH218743** |
|  | *E. armeniacum* (MWC 1229) | SC | Present (this study) | AF263748.1 | AF518058.1 | AF266993.1 |  | KR908829.1 | AF260165.1 |
|  | *E. armeniacum* (F. Pinheiro 651 – SP) | AN | Present (this study) | - | - | - | - | - | - |
|  | *E. campestre* (F. Pinheiro 607 - SP) | SC, AN | Absent (this study) | - | - | JQ646009 | JQ645989 | JQ645969 | **MH218741** |
|  | *E. cardiochilum* | SC | Present (Hágsater & Soto 2003) | FJ238563.1 | - | - | - | FJ238584.1 | - |
|  | *E. centropetalum* (MWC 6084) | SC | Present (Hágsater & Sánchez-Saldana 2006) | AF263782.1 | JQ593047.1 | AF267003.1 | - | KR908857.1 | AF260175.1 |
|  | *E. chlorocorymbos* (A. Karremans 1627) | SC | Present (Hágsater & Sánchez-Saldana 2009) | KM495154.1 | - | - | - | - | - |
|  | *E. ciliare* (F. Pupulin 4523) | SC | Absent (this study) | FJ238564.1 | - | - | - | FJ238583.1 | KM485134.1 |
|  | *E. ciliare* (F. Pinheiro 652 – SP) | AN | Absent (this study) |  | - | - | - | - | - |
|  | *E. cocoense* (JBL-S392) | SC | Present (Hágsater et al. 1999) | EU214345.1 | - | - | - | - | - |
|  | *E. coronatum* (F. Pinheiro 647 – SP)) | AN | Absent (this study) | - | - | - | - | - | - |
|  | *E. cristatum* (F. Pinheiro 573 - SP) | SC, AN | Present (this study) | - | - | FJ869396.1 | - | - | - |
|  | *E. densiflorum* (F. Pinheiro 649 – SP) | AN | Present (this study) | - | - | - | - | - | - |
|  | *E. diffusum* | SC | Present (Hágsater & Sánchez-Saldana 2008) | FJ238565.1 | - | - | - | FJ238585.1 | FJ238549.1 |
|  | *E. falcisepalum* | SC | Absent (Marques et al. 2014) | KF679552.1 | KF679628.1 | KF715840.1 | - | - | - |
|  | *E. goniorhachis* (FP 5358) | SC | Present (Hágsater & Sánchez-Saldana 2007) | EU214347.1 | - | - | - | - | - |
|  | *E. hunterianum* (FP 5384) | SC | Present (Hágsater & Sánchez-Saldana 2006) | EU214348.1 | - | - | - | - | - |
|  | *E. insulanum* (JBL-S391) | SC | Present (Hágsater & Sánchez-Saldana 2007) | EU214349.1 | - | - | - | - | - |
|  | *E. jimenezii* (JBL-S389) | SC | Present (Hágsater et al. 1999) | EU214350.1 | - | - | - | - | - |
|  | *E. madsenii* | SC | Present (Hágsater et al. 1999) | KF679524.1 | KF679600.1 | KF715759.1 | - | - | - |
|  | *E. medusae* | SC | Present (Hágsater et al. 1999) | EF079313.1 | - | - | - | - | EF079382.1 |
|  | *E. miserrimum* (DB 1804) | SC | Present (Hágsater & Sánchez-Saldana 2007) | EU214351.1 | - | - | - | - | - |
|  | *E. nocturnum* (D. Bogarin 2250 – JBL) | SC | Present (this study) | KM495141.1 | - | JQ646022.1 | JQ646002.1 | JQ645982.1 | AY008514.1 |
|  | *E. nocturnum* (F. Pinheiro 620 – SP) | AN | Present (this study) |  | - | - | - | - | - |
|  | *E. odontochilum* (DB 2262) | SC | Present (Hágsater & Sánchez-Saldana 2007) | EU214352 | - | - | - | - | - |
|  | *E. parkinsonianum* | SC | Absent (Hágsater & Sánchez-Saldana 2010) | FJ238566.1 | - | - | - | FJ238586.1 | FJ238550.1 |
|  | *E. polyanthum* | SC | Present (Hágsater & Sánchez-Saldana 2008) | - | - | - | - | FJ238587.1 | FJ238551.1 |
|  | *E. pseudepidendrum* | SC | Present (Hágsater & Sánchez-Saldana 2008) | EU214167.1 | - | AF266986.1 | - | - | EU554346.1 |
|  | *E. raniferum* | SC | Present (Hágsater & Sánchez-Saldana 2008) | FJ238567.1 | - | - | - | FJ238588.1 | FJ238552.1 |
|  | *E. rhopalostele* | SC | Present (Hágsater & Sánchez-Saldana 2001) | KF679542.1 | KF679618.1 | KF715812.1 | - | - | KC165028.1 |
|  | *E. rigidum* | SC | Absent (this study) | EF079311.1 | - | - | - | - | - |
|  | *E. rigidum* (F. Pinheiro 649 – SP) | AN | Absent (this study) | - | - | - | - | - | - |
|  | *E. robustum* (F. Pinheiro 645 - SP) | SC, AN | Absent (this study) | **MH218779** | - | **MH218800** | - | - | **MH218744** |
|  | *E. schlechterianum* (MWC 301) | SC | Present (Hágsater & Sánchez-Saldana 2007) | EF079314.1 | - | AF267000.1 | - | - | EF079381.1 |
|  | *E. smaragdinum* (F. Pinheiro 577 - SP) | SC | Present (Garay & Dunsterville 1972) | - | - | FJ869417.1 | - | - | - |
|  | *E. tridactylum* (F. Pinheiro 646 - SP) | PC, SC | Absent (Pansarin & Pansarin (2014) | **MH218777** | **MH218780** | **MH218802** | - | - | **MH218745** |
|  | *E. vesicatum* (F. Pinheiro 643 - SP) | SC, AN | Present (this study) | **MH218778** | - | **MH218799** | - | - | **MH218742** |
|  | *E. viviparum* (F. Pinheiro 650 – SP) | AN | Present (this study) | - | - | - | - | - | - |
| Laeliinae genera outgroups | *Arpophyllum giganteum* | PC, SC | Present (Pridgeon et al. (2005) | AF263625.1 | AF074110.1 | AF266975 | - | - | AF266742.1 |
|  | *Cattleya lundii* (Brieger Coll. 30692) | SC | Absent (Pridgeon et al. 2005) | EU140013.1 | - | JN600706.1 | JN600838.1 | JN600779.1 | AY008645.1 |
|  | *C. porphyroglossa* (van der Berg s.n. HUEFS) | SC | Absent (Pridgeon et al. 2005) | EU139980.1 | - | JN600698.1 | JN600832.1 | JN600771.1 | JN600951.1 |
|  | *C. violacea* (Brieger Coll. 28495) | PC, SC | Absent (Pridgeon et al. 2005) | AY396102.1 | AF518071.1 | AF267039.1 | JN600836.1 | JN600777.1 | AF260206.1 |
|  | *Encyclia oncidioides* (MWC5938) | PC, SC | Absent (Pridgeon et al. 2005) | AF263788.1 | AF518062.1 | AF267013.1 | - | - | AF260184.1 |
|  | *Encyclia tampensis* | SC | Absent (Pridgeon et al. 2005) | AY396116.1 | KJ773476.1 | AY422419.1 | - | - | AY429401.1 |
|  | *Meiracyllium trinasutum* | PC, SC | Present (Pridgeon et al. 2005) | AY396090.1 | AF074192.1 | AY422393.1 | - | - | EF079372.1 |
| Subfamily Epidendroidae | *Palmorchis trilobulata* | PC | - | AJ310052.1 | AF074206.1 | - | - | - | - |
|  | *Sobralia macrantha* | PC | - | AF263681.1 | AF074228.1 | - | - | - | - |
|  | *Listera ovata* | PC | - | AF263668.1 | KM360858.1 | - | - | - | - |
|  | *Epipactis royleana* | PC | - | KF419098.1 | KF419093.1 | - | - | - | - |
| Higher Epidendroids | *Masdevallia uniflora* | PC | - | AY368416.1 | AF518040.1 | - | - | - | - |
|  | *Restrepia xanthophthalma* | PC | - | AY370654.1 | AY370653.1 | - | - | - | - |
|  | *Isochilus amparoanus* | PC | - | AY370654.1 | AF518031.1 | - | - | - | - |
|  | *Eria ferruginea* (sp0959) | PC | - | KX526717.1 | AF074164.1 | - | - | - | - |
|  | *Calanthe tricarinata* (J.W. Zhai 140) | PC | - | KF673821.1 | KF852748.1 | - | - | - | - |
|  | *Wullschlaegelia aphylla* | PC | - | AY368434.1 | AY368436.1 | - | - | - | - |
|  | *Pleione formosona* | PC | - | AF302705.1 | AF264173.1 | - | - | - | - |
|  | *Galeandra devoniana* | PC | - | KF660268.1 | AF074171.1 | - | - | - | - |
|  | *Mormodes* sp. (KMC1998) | PC | - | AY368417.1 | AF074196.1 | - | - | - | - |
|  | *Oncidium andradeanum* (Williams N079 FLAS) | PC | - | FJ563866.1 | FJ534235.1 | - | - | - | - |
|  | *Stanhopea ecornuta* | PC | - | AY368430.1 | AF074230.1 | - | - | - | - |
|  | *Zygopetalum intermedium* | PC | - | AF263689.1 | AF074246.1 | - | - | - | - |
|  | *Lycaste cruenta* | PC | - | AF239438.1 | AF074185.1 | - | - | - | - |
|  | *Maxillaria cucullata* | PC | - | DQ209871.1 | AF074190.1 | - | - | - | - |
|  | *Bifrenaria harrisoniae* | PC | - | AY368394.1 | AF074112.1 | - | - | - | - |
| External group | *Codonorchis lessonii* | PC | - | DQ414993.1 | AJ542399.1 | - | - | - | - |
|  | *Disa glandulosa* | PC | - | AF263654.1 | AF274006.1 | - | - | - | - |
|  | *Habenaria quinqueseta* | PC | - | KJ021406.1 | KJ773544.1 | - | - | - | - |
|  | *Orchis mascula* | PC | - | AY368385.1 | KM360905.1 | - | - | - | - |
|  | *Pterostylis curta* | PC | - | EF079298.1 | AJ542400.1 | - | - | - | - |
|  | *Megastylis glandulosa* | PC | - | AJ543950.1 | AJ542401.1 | - | - | - | - |
|  | *Altensteinia fimbriata* (ABA 2726 NY) | PC | - | EF065583.1 | FJ571315.1 | - | - | - | - |
|  | *Chloraea prodigiosa* (CONC M.A. Cisternas 101) | PC | - | GQ917043.1 | FR831982.1 | - | - | - | - |
|  | *Sarcoglottis acaulis* | PC | - | EU395447.1 | AJ542424.1 | - | - | - | - |
|  | *Microtis parviflora* | PC | - | AF263671.1 | AF074194.1 | - | - | - | - |
|  | *Diuris sulphurea* | PC | - | AF263655.1 | AJ542398.1 | - | - | - | - |
|  | *Pachyplectron arifolium* (TJM 1171 NY) | PC | - | AJ310051.1 | FJ571336 | - | - | - | - |
|  | *Goodyera pubescens* | PC | - | AF263663.1 | AF074174 | - | - | - | - |
|  | *Platythelys querceticola* | PC | - | AY368386.1 | AF074216.1 | - | - | - | - |
|  | *Ludisia discolor* | PC | - | AJ543911.1 | AJ542395.1 | - | - | - | - |
|  | *Dossinia marmorata* | PC | - | AJ543947.1 | AJ542405.1 | - | - | - | - |

**Literature Cited in Supplementary Table S1**

**Hágsater E, Sánchez-Saldana LM. 2007.** *Icones Orchidacearum: fascicle 9*. México: Herbario AMO.

**Hágsater E, Soto M. 2003.** *Icones Orchidacearum: fascicles 5 and 6*. México: Herbario AMO.

**Hágsater E, Sánchez-Saldana LM.** **2006.** *Icones Orchidacearum: fascicle 8*. México: Herbario AMO.

**Hágsater E, Sánchez-Saldana LM.** **2009.** *Icones Orchidacearum: fascicle 12*. México: Herbario AMO.

**Hágsater E, Sánchez-Saldana LM, García-Cruz CJ. 1999.** *Icones Orchidacearum: fascicle 3*. México: Herbario AMO.

**Hágsater E, Sánchez-Saldana LM.** **2008.** *Icones Orchidacearum: fascicle 11*. México: Herbario AMO.

**Marques I, Draper D, Riofrío L, Naranjo C. 2014.** Multiple hybridization events, polyploidy and low postmating isolation entangle the evolution of neotropical species of *Epidendrum* (Orchidaceae). *BMC Evolutionary Biology* **14**: 20.

**Hágsater E, Sánchez-Saldana LM. 2010.** *Icones Orchidacearum: fascicle 13*. México: Herbario AMO.

**Hágsater E, Sánchez-Saldana LM.** **2001.** Icones Orchidacearum: fascicle 4. México: Herbario AMO.

**Garay LA, Dunsterville GCK. 1972.** *Venezuelan orchids illustrated vol. 5*. London: Andre Deutsch.

**Pansarin ER, Pansarin LM. 2014.** Reproductive biology of *Epidendrum tridactylum* (Orchidaceae: Epidendroideae): a reward-producing species and its deceptive flowers. *Plant Systematics and Evolution* **300**: 321-328.

**Pridgeon AM, Cribb PJ, Chase MW, Rasmussen F. 2005.** *Genera Orchidacearum: Volume 4. Epidendroideae (Part One)*. Oxford: Oxford University Press.
